# Immunohistochemical Expression of Upregulated Gene 4 Protein Expression (URG4/URGCP) and Its Association with 5-Year Survival in Patients with Colon Adenocarcinoma

**DOI:** 10.3390/jcm12175477

**Published:** 2023-08-23

**Authors:** Marlena Brzozowa-Zasada, Adam Piecuch, Marek Michalski, Katarzyna Stęplewska, Natalia Matysiak, Marek Kucharzewski

**Affiliations:** 1 Department of Histology and Cell Pathology in Zabrze, Faculty of Medical Sciences in Zabrze, Medical University of Silesia in Katowice, 40-055 Katowice, Poland; 2 Department of Pathology, Institute of Medical Sciences, University of Opole, 45-052 Opole, Poland; 3 Faculty of Health Sciences, Jan Dlugosz University of Czestochowa, 42-200 Czestochowa, Poland

**Keywords:** URG4/URGCP, colon adenocarcinoma, colon cancer, oncology, immunohistochemistry, prognostic factors, 5-year survival rate

## Abstract

(1) Background: Colorectal cancer (CRC) is the third most common cancer in terms of incidence and mortality. Approximately 90% of all colorectal cancer cases are adenocarcinomas, originating from epithelial cells of the colorectal mucosa. Upregulated gene 4 (URG4) is an oncogene involved in cancer development. The aim of the study was to assess the immunohistochemical expression of URG4 protein expression in Polish patients with colon adenocarcinoma who were not treated with any therapy before radical surgery. (2) Methods: The study used colon tissue samples taken from people with a confirmed diagnosis of colorectal adenocarcinoma after a thorough histopathological examination. The associations between the immunohistochemical expression of URG4 and clinical parameters were analyzed by the Chi2 test or Chi2Yatesa test. The study conducted an analysis of the correlation between the expression of URG4 and the five-year survival rate of patients through the application of the Kaplan–Meier analysis and the log-rank statistical test. The intracellular localization of URG4 was identified through the utilization of transmission electron microscopy (TEM) methodology. (3) Results: In univariate Cox regression analyses, immuno-histochemical expression of URG4, grade of histological differentiation, depth of invasion, angioinvasion, PCNA expression, stage of disease and lymph node involvement were found to be significant prognostic factors. Within our patient cohort, it was observed that the degree of tumour differentiation and URG4 expression were found to be distinct prognostic factors in regard to the 5-year survival rates of those with colon adenocarcinoma. (4) Conclusions: High immunohistochemical expression of URG4 correlates with poor prognosis in patients with colon adenocarcinoma.

## 1. Introduction

Colorectal cancer (CRC) is the third most common cancer in terms of both incidence and mortality, with rates of 6.1% and 92%, respectively. It is predicted that there will be a significant increase in mortality from rectal cancer by 2035, with a predicted increase of 60%. There will also be an increase in the mortality rate from colon cancer, with a projection of 71.5% [1]. These figures are likely to vary from one country to another, depending on the level of economic development. The disease is thus considered to be an indicator of countries’ socioeconomic development [2]. It has been reported that the survival rate of colorectal cancer depends on the stage at which it is diagnosed, with those diagnosed at a later stage having a worse chance of surviving [3]. The 5-year survival rate for colorectal cancer diagnosed at an early stage is 90 percent, compared with 13 percent for those diagnosed later [4,5].

Approximately 90% of malignancies that occur in the colon and rectum are classified as adenocarcinomas. Adenocarcinomas are known to arise from the epithelial cells that make up the lining of the colon. Less common types of colorectal cancer include neuroendocrine carcinoma, squamous cell carcinoma, adenosquamous carcinoma, spindle cell carcinoma and undifferentiated carcinoma. The typical manifestation of adenocarcinoma is characterised by the presence of glandular cells, which serve as the basis for the histological evaluation of the tumour. In the clinical setting, the majority of cases of colorectal adenocarcinoma are often found to be moderately differentiated. According to the cited source [6], a significant proportion of cases, specifically 10% and 20%, are characterised as having well and poorly differentiated features, respectively. A large number of research initiatives have been undertaken to evaluate the prognostic potential of various molecular markers in colorectal cancer [7,8,9]. However, despite the high number of potential biomarkers for clinical use in patients with this complex and heterogeneous disease, few have received approval for use in practice. Furthermore, prediction of prognosis and response to treatment remain a major therapeutic challenge [8].

Upregulated gene 4 (URG4), also called an upregulator of cell proliferation (URGCP), is a 3607 kb mRNA oncogene located on chromosome 7p13 that has been implicated in the progression of several tumours, including hepatocellular cancer [10], ovarian cancer [11], gastric cancer [12], bladder cancer [13], glioblastoma [14], non-small cell lung cancer [15], medullary thyroid cancer [16], prostate cancer [17] and leukaemia [18]. The overexpression of URG4 not only results in the progression of the tumour but also leads to the development of metastases and disease recurrence during its course [19].

The current research indicates a lack of information regarding the clinical utility of URG4 expression in patients diagnosed with colorectal cancer in Europe, particularly in cases of colon adenocarcinoma. In view of the above, we have carried out a study of the expression of the URG4 protein in a cohort of European (specifically Polish) patients diagnosed with colon adenocarcinoma who have not received any radiotherapy or chemotherapy prior to surgical intervention. URG4 protein expression was found to be significantly correlated with clinicopathological factors. In addition, a correlation between URG4 expression and proliferating cell nuclear antigen (PCNA) expression was observed. The critical function of PCNA in promoting cell proliferation has established its frequent use as a marker of tumour proliferation [20,21]. The aim of the present study was to evaluate the prognostic potential of the URG4 protein, a clinically significant factor in oncology, with respect to patient survival over a five-year period. In addition, the intracellular distribution of URG4 within the cellular composition of cancer tissues was investigated. This was achieved using immunogold labelling.

## 2. Materials and Methods

### 2.1. Patients and Tumour Samples

In the current study, samples of colon tissue were obtained from patients with established colon adenocarcinoma, confirmed by histopathological examination, who underwent colon resection procedures at Jaworzno Municipal Hospital between January 2014 and December 2015. Patients who received preoperative radiotherapy or chemotherapy, had severe medical problems or distant metastases, underwent resection for tumour recurrence, had inflammatory bowel disease-related colorectal cancer, or had a histopathological subtype other than colon adenocarcinoma were excluded from the study.

According to a standardised protocol, histopathological sections were obtained from each surgical specimen, consisting of tumour fragments and parts of adjacent tissue without tumour abnormalities. The specimens were fixed in formalin and embedded in paraffin blocks. Paraffin blocks were then cut, and sections were routinely H&E-stained for histopathological diagnosis. Marginal tissue sections were also examined. If any cancer cells were detected, the material was excluded from the study. To evaluate the prognostic significance of the URG4 protein, patients were followed for 5 years to evaluate 5-year survival.

### 2.2. Immunohistochemical Staining

Paraffin-embedded tissue blocks containing formalin-preserved colon adenocarcinoma specimens were cut into 4-micron-thick sections. The tissue sections were placed on polysine-coated slides, subjected to xylene for deparaffinisation and hydrated through a series of graded concentrations of alcohol. For the retrieval of antigenicity, the sections were treated with microwaves. Sections were then incubated with antibodies against URG4 (RayBiotech polyclonal antibody, code 102-18472, final dilution 1:500, Raybiotech Life, Inc. 3607 Parkway Lane Suite 200; Peachtree Corners, GA 30092, USA) and PCNA (polyclonal antibody from GeneTex. Cat. No. GTX100539, final dilution 1:600, Irvine, CA, USA). To visualise protein expression, sections were developed using the BrightVision detection system and Permanent AP Red Chromogen. The nuclei were counterstained using Mayer’s haematoxylin for the study. In addition, sections of healthy mucosa from patients who had underwent a screening colonoscopy and were free of inflammatory or cancerous lesions were analysed for the expression of URG4 and PCNA. The intensity was graded as follows: 0, no signal; 1, weak; 2, moderate; and 3, strong staining. The frequency of positive cells was semiquantitatively determined by assessing the whole section and each sample was scored on a scale from 0 to 4: 0, negative; 1, positive staining in 10 to 25% of cells; 2, 26 to 50% of cells; 3, 51 to 75% of cells; and 4, 76 to 100% of cells. Finally, a total score from 0 to 12 was calculated and graded as follows: I, score 0 to 1; II, 2 to 4; III, 5 to 8; IV, 9 to 12. Grade I was considered negative and grades II, III and IV were considered positive. Grades I and II represented no or weak staining (low expression) and grades III and IV represented strong staining (high expression).

The grading was carried out by two pathologists independent of each other. Differences were the subject of discussion until a consensus had been reached.

### 2.3. Statistical Analysis

In the present study, an analysis of the relationship between the immunohistochemical expression of URG4 and relevant clinical parameters was performed using the Statistica 9.1 software package developed by StatSoft, Krakow, Poland. For the evaluation of all numerical variables, the statistical measures of median and range were used. Both the chi-squared test (Ch2 test) and Yates’ chi-squared test (Chi2Yatesa test) were used to assess the relative characteristics of the studied groups. The aim of the present study was also to investigate the possible association between the intensity of URG4 expression and patient survival. The relationship between the intensity of URG4 expression and the 5-year survival of patients was tested using Kaplan–Meier analysis and log-rank test. The results were considered to be statistically significant if *p* < 0.05.

### 2.4. Immunogold Electron Microscopy

In the present study, samples were immobilised in a 4% solution of paraformaldehyde in 0.1 M phosphate-buffered saline (PBS) at room temperature for two hours, followed by several rinses in PBS. Following washing, the specimens were dehydrated in a graded ethanol series and infiltrated for 30 min on ice in a 2:1 (*v*:*v*) ethanol/LR White mixture and a 1:2 (*v*:*v*) mixture. After then, the samples were infiltrated with pure LR White. An RMC Boeckeler Power Tomo PC ultramicrotome with a diamond blade (45°; Diatom AG, Biel, Switzerland) was used to cut ultrathin sections (70 nm). Ultrasections were immunolabeled and placed on Formvar-coated 200 mesh nickel grids. Sections on the grids were pre-incubated for 30 min by floating on drops of 50 mM NH4 Cl in PBS, followed by 30 min of blocking on drops of 1% BSA in PBS. The grids were then treated overnight (16–18 h) at 4 °C with a 1:20 dilution of primary anti-URG4 antibody in BSA. By incubating the sections for 1 h on immunogold conjugated goat anti-mouse IgG 15 nm (BBInternational BBI Solutions, Sittingbourne, UK) diluted 1:100, the bound antibodies were localized. Finally, before staining with 0.5% aqueous uranyl acetate, the grids were rinsed with PBS drops (five changes, 5 min each) and water (three changes, 3 min each). The main antibody was not used in the controls. The grids were then air-dried before being examined at 120 kV in a TECNAI 12 G2 Spirit Bio Twin FEI Company trans-mission electron microscope. Morada CCD camera (Gatan RIO 9, Pleasanton, CA, USA) was used to collect the images.

## 3. Results

### 3.1. Characteristics of the Patients

The sample consisted of 70 male and 68 female participants with a mean age of 65 years and an age range ranging between 56 and 77 years. The occurrence of malignancies was determined to be located in the right colon in 72 individuals, which comprises 52.17% of the patient cohort, while in the left colon, it is observed in 66 cases, equivalent to 47.83% of the total cases. The specimens under study were categorized into three distinct histological differentiation grades denoted as G1, G2, and G3. The distribution of the cases based on the differentiation grade was as follows: G1—32 cases (23. 19%), G2—67 cases (48.55%), and G3—39 cases (28.26%), as summarized in Table 1.

**Figure 1:**
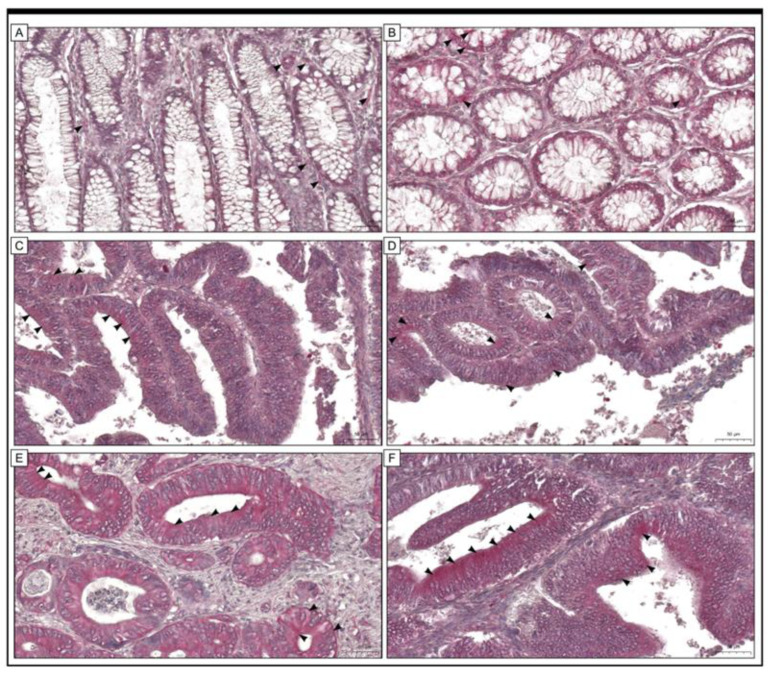
The expression of URG4 in colon adenocarcinoma tissue (**C**–**F**) and adjacent non-cancerous tissue margins (**A**,**B**) is shown in this study by photomicrographs. (**A**,**B**) Low levels of immunohistochemical reactivity were detected in cells found within the tissue margin of the colonic mucosa of non-pathological specimens. The present study has demonstrated that URG4 expression (black arrowheads) in colon adenocarcinoma specimens can be classified into two distinct groups, namely low (**C**,**D**) and high (**E**,**F**). The scale bar is 50 µm (**A**–**F**).

The study population comprised 138 patients, of whom 33 (23.91%) were diagnosed with stage I disease, 32 (23.19%) with stage II disease and 73 (52.90%) with stage III disease. Examination of the colon adenocarcinoma specimens revealed a distinct immunohistochemical response. URG4 protein was found in the cytoplasm and plasma membrane of both stromal and malignant cells. Cells in non-pathological colon tissue were also stained. The results of the study show a remarkable observation: a significant proportion of colon adenocarcinoma tissue showed a robust level of expression. In contrast, the level of expression was reported to be low in the cells of the adjacent non-pathological colonic mucosa. This is shown in Figure 1.

### 3.2. The Relationship between the Immunohistochemical Expression of URG4 and the Most Important Clinical Features of the Patients

**Figure 2:**
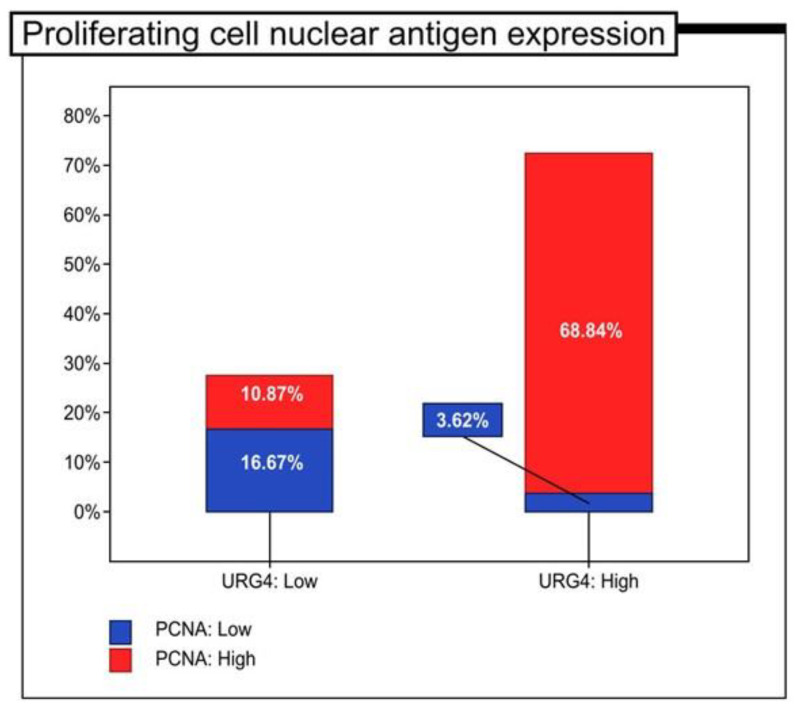
The proportion of patients with colon adenocarcinoma with high and low immunohistochemical expression of proliferating cell nuclear antigen (PCNA) (*n* = 138).

In the present study cohort, 100 (72.46%) colon adenocarcinoma specimens dis-played high immunohistochemical expression of URG4 protein. 38 (27.54%) specimens demonstrated low immunoreactivity. Next, the findings of the immunohistochemical analysis were related to patient clinicopathological characteristics and 5-year survival. The URG4 expression was associated with the histological grade of the tumour (*p* < 0.001, Chi2 test). A high level of URG4 protein expression was detected in 8 (25%), 55 (82.09%) and 37 (94.87%) of the G1, G2 and G3 tumours, respectively. In 24 (75%), 12 (17.91%) and 2 (5.13%) of the G1, G2 and G3 tumours, respectively, a low level of immunohistochemical URG4 protein expression was observed. URG4 expression was additionally related to PCNA immunohistochemistry (*p* < 0.001 Chi2Yatesatest). In patients with low PCNA ex-pression, 23 (16.67%) showed low URG4 expression and 5 (3.62%) showed high URG4 expression. In contrast, in patients with high PCNA expression, 15 (10.87%) demonstrated low URG4 immunohistochemical expression and 95 (68.84%) revealed high expression of this protein (Table 2, Figure 2).

The association between URG4 expression and angiogenesis (*p* < 0.05) is remarkable and worth considering (*p* < 0.001; Chi2 test). A study performed on a cohort of patients showed that a significant percentage of individuals, namely, 34.21%, showed high URG4 immunohistochemical expression in the absence of angioinvasion. The remaining 65.79% showed low levels of immunoreactivity. In contrast, the results showed that 87% of patients with positive angioinvasion expressed high levels of URG4, while 13% of patients had low levels of URG4 immunoreactivity. The immunohistochemical expression of URG4 showed a significant correlation with the depth of invasion (T) based on the chi-square test that was performed (*p* < 0. 001). Within the cohort of patients classified as T1/T2, it was observed that 18 individuals (43.90%) demonstrated an increased immunohistochemical response, while 23 patients (56.10%) showed a lower degree of expression. The present study reports a remarkable immunohistochemical response to URG4 in a sample of 82 patients (84.54%) diagnosed with T3/T4 tumours. A decreased level of expression was observed in 15 patients (15.46%). In the cohort of individuals diagnosed with stage I pathology, 16 (48.48%) showed increased URG4 expression levels while 17 (51.52%) showed decreased expression levels. Among patients diagnosed with stage II disease, the majority (84.38%) were characterised by high levels of immunoreactivity, while a minority (15.63%) showed low levels. In contrast, among patients diagnosed with stage III disease, 57 (78.08%) showed strong immunoreactivity, while 16 (21.92%) showed low levels of immunoreactivity. This finding was found to be statistically significant, with a *p*-Value of 0.002, as determined by a chi-squared test (Table 3).

### 3.3. The Prognostic Role of URG4 Expression in Association with 5-Year Survival

**Figure 3:**
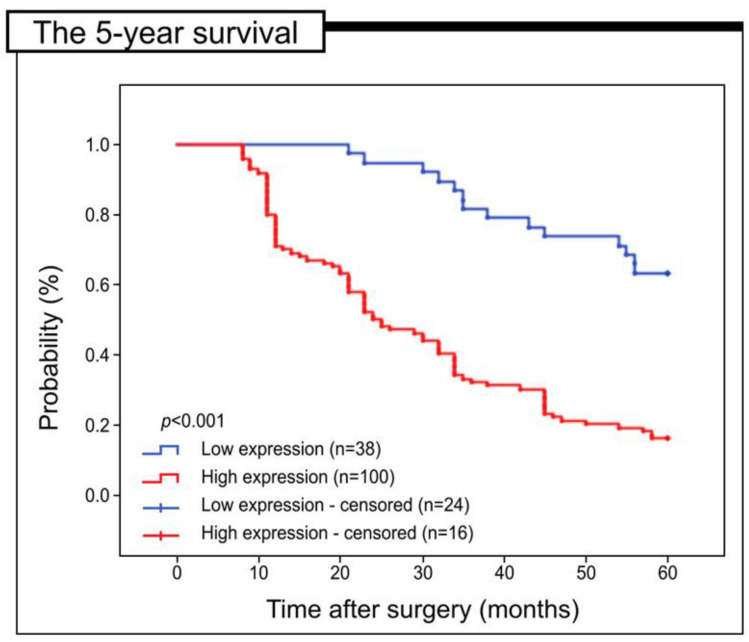
Kaplan-Meier curves of data from univariate analysis (log-rank test) indicating the 5-year survival rate for patients with high versus low levels of URG4 immunohistochemical expression.

In order to determine the prognostic value of URG4 expression in colorectal cancer patients, a study was performed to investigate its potential association with 5-year survival, using Kaplan–Meier survival curves for all samples. The statistical analysis shows that the group with low levels of URG4 has a notable discrepancy in the 5-year survival rate, as determined by a log-rank test with a *p*-value of less than 0.05.001), which has significant implications (Figure 3).

**Figure 4:**
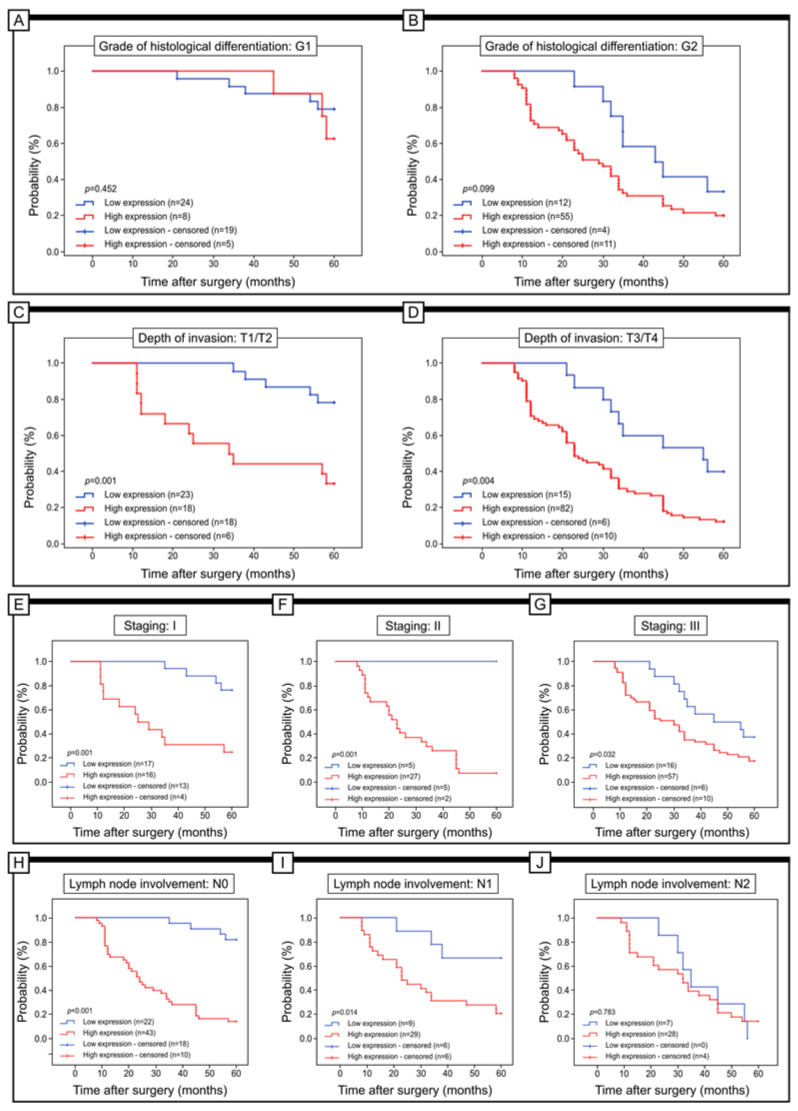
Kaplan–Meier curves of univariate analysis data (log-rank test) of patients with high versus low levels of URG4 immunohistochemical expression. (**A**,**B**) Five-year survival of patients with G1 (**A**) and G2 (**B**) grade of differentiation; with T1/T2 (**C**) and T3/T4 depth of invasion (**D**); with staging I (**E**), staging II (**F**), and staging III (**G**); without lymph node involvement (N0) (**H**) and with N1 lymph node involvement (**I**), and with N2 lymph node involvement (**J**).

Moreover, an assessment was conducted on the relevance of URG4 expression for the 5-year survival of patients classified into distinct subgroups based on factors such as the degree of histological differentiation, depth of invasion, staging, lymph node involvement, and PCNA expression (see Figure 4). Specifically, the expression of URG4 did not display any significant correlation with the 5-year survival of patients who were placed in G1 (log-rank test, *p* = 0. 452), G2 (log-rank test, *p* = 0. 099), and G3 (log-rank test, *p* = 0. 005). In patients with T1/T2 invasion depth, those with low levels of URG4 immunohistochemistry had a significantly longer 5-year survival than those with high levels of URG4 (log-rank test, *p* < 0.011). Patients with T3/T4 depth of invasion had similar outcomes (log-rank test, *p* = 0.004). In addition, a low level of URG4 expression was associated with 5-year survival in patients with stage I and II of the disease (log-rank test, *p* < 0.001). In patients with stage III disease, a low level of URG4 expression was also associated with 5-year survival (log-rank test, *p* = 0.032). In patients without lymph node involvement (N0), a low level of URG4 expression was also associated with better survival (log-rank; *p* < 0.001). In patients with lymph node involvement (N1 or N2), better survival was also connected with a low level of URG4 immunohistochemical expression in cancer tissues; *p* = 0.014 and *p* = 0.783 respectively (Figure 4).

**Figure 5:**
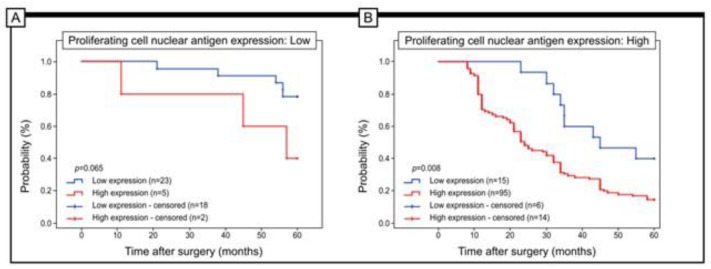
Kaplan–Meier curves of univariate analysis data (log-rank test) of patients with high versus low levels of URG4 immunohistochemical expression. (**A**,**B**) Five-year survival of patients with low (**A**) and high (**B**) expression of PCNA.

Low URG4 expression was related to good prognosis in patients with both high and low PCNA immunohistochemical expression (log-rank test, *p* = 0.001) (Figure 5).

In univariate Cox regression analyses, immunohistochemical expression of URG4, grade of histological differentiation, depth of invasion, angioinvasion, PCNA expression, stage of diseases and lymph node involvement were found to be significant prognostic factors. In our cohort of patients, the grade of tumour differentiation and the expression of URG4 were independent prognostic factors for the 5-year survival of patients with colorectal adenocarcinoma (Table 4).

### 3.4. Detection of URG4 at the Cellular Level by the Use of TEM

**Figure 6:**
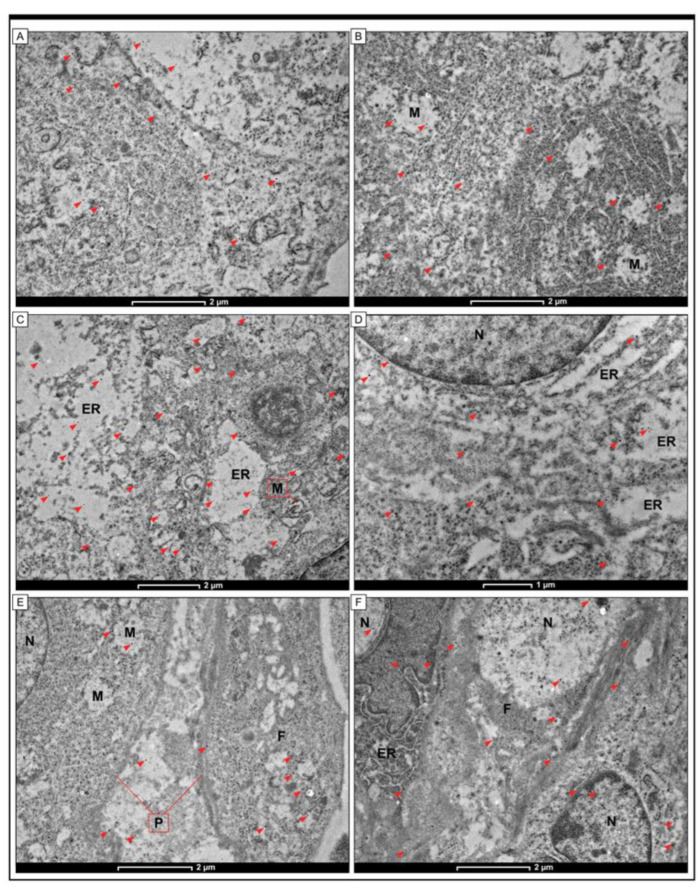
Detection of URG4 protein by immunogold labelling in cells of colon adenocarcinoma tissue. The small electron-dense granules (red arrowheads) were found in cells of non-pathological colon tissue (**A**,**B**) and cells of colon adenocarcinoma samples (**C**,**D**). The black electron-dense granules were also found in fibroblasts in the tumour stroma compartment (**E**,**F**). A small number of electron-dense granules were also detected in the plasma membrane and within the cisterns of the endoplasmic reticulum in cells from non-pathological colon mucosa. Moreover, those granules were also visible in mitochondria (M) and cytoplasm. In cancer cells, the localisation of URG4 was similar to that in non-pathological cells. URG4 was detected within the mitochondria (M) and endoplasmic reticulum (ER). In some cells, the granules were also found in the membrane of the nuclear envelope (N). In stromal fibroblasts, gold granules were associated with the plasma membrane (P), mitochondria (M) and small vesicles detected within the cytoplasm. Some cells also showed granules near the intermediate filament bundles (**F**). The scale bar is 1 µm (**D**), 2 µm (**A**–**C**,**E**,**F**).

A technique using immunogold labelling was implemented in the study to reveal the location of the URG4 protein in colon adenocarcinoma specimens. A limited quantity of electron-dense granules was identified within the plasma membrane and the cisterns of the endoplasmic reticulum in the cells derived from the non-pathological colon mucosa. In addition, these granules could also be seen in the mitochondria and in the cytoplasm. In cancer cells, the localisation of URG4 was similar to the localisation of URG4 in non-pathological cells. URG4 was found in the mitochondria and in the endoplasmic reticulum. In some cells, granules were found in the nuclear envelope membrane. In stromal fibroblasts, gold granules were associated with the plasma membrane, mitochondria and small vesicles that were detected in the cytoplasm. Granules close to intermediate filament bundles were also observed in some cells (Figure 6).

## 4. Discussion

Oncogenes, which drive the process of cancer development, are mutated forms of normal cellular genes known as proto-oncogenes. Proto-oncogenes are well-conserved throughout their evolution and usually regulate fundamental cellular processes such as the development and differentiation of cancer cells [22]. Oncogenes encode proteins that play crucial roles in cell proliferation, apoptosis, and cell-cycle control. URG4 is a transcriptional activator downstream gene of hepatitis B virus X protein (HBx), named for its up-regulation of cell proliferation [23]. URG4 expression is mainly regulated at the transcriptional level by DNA methylation, transcription factors and drugs [24]. As revealed by the studies, URG4 has been implicated in the initiation, development, treatment and prognosis of several cancers, e.g., nasopharyngeal carcinoma [25], cervical cancer [26] and gastric cancer [12]. We, therefore, investigated whether it is also associated with the development and progression of colon adenocarcinoma. The results of our study showed that approximately 72% of colorectal adenocarcinoma specimens had high levels of URG4 protein expression, while only 28% had low levels of immunoreactivity. The expression of URG4 was found in the plasma membrane and in the cytoplasm, particularly in the ER and in the mitochondria. The studies showed that the localisation of URG4 expression is different in different tumours. In hepatocellular carcinoma [15] and ovarian cancer [27], URG4 expression is associated with the cytoplasm. In contrast, in cervical cancer and nasopharyngeal carcinoma, URG4 protein was located at the plasma membrane [25,26].

The findings of the statistical analysis indicated a noteworthy correlation between elevated levels of URG4 immunohistochemical expression and the histological grading of the tumour (*p* < 0.001, Chi-squared test), depth of invasion (*p* < 0.001, Chi-squared test), angioinvasion (*p* < 0.001, Chi-squared test). and staging (*p* = 0.002, Chi-squared test). Hoverwer, in this case, URG4 expression is numerically lower in stage III vs. stage II (84 vs 78%). The findings of the study suggest that a substantial increase in URG4 protein expression is evident only in a relatively small proportion of G1 tumours, but is significantly higher in G2 tumours (reaching 82%) and peaks in G3 tumours (achieving 95%). Forty-eight percent of patients diagnosed with stage I disease displayed elevated levels of URG4 expression while a significant majority of patients (84%) with stage II disease manifested high levels of URG4 immunohistochemical expression. In patients diagnosed with stage III disease, approximately 78% demonstrated high expression of URG4 protein. In the present context, the finding that the expression pattern of URG4 exhibited a significant correlation with the immunohistochemical expression of PCNA (*p* < 0.001) is notable. Based on the study, a considerable percentage of patients exhibited a decrease in URG4 expression along with a reduction in PCNA expression, with 82% of cases showing this correlation. In contrast, URG4 expression levels were only elevated in 17% of the patients. In contrast, a mere 14% of patients exhibiting elevated levels of PCNA also demonstrated diminished expression of URG4. In contrast, patients from the former category showed a significant increase in URG4 expression, amounting to 86%. Regarding URG4 expression, a statistically significant difference in survival was observed between patients exhibiting low versus high PCNA expression. The study observed a correlation between low levels of PCNA expression and increased 5-year survival rates in patients, which was further linked with decreased levels of URG4 immunohistochemical expression. The present study reveals that the enhanced survival rate observed in patients exhibiting a high level of PCNA was eliminated upon assessment of low expression levels of URG4 within this subgroup (log rank test *p*-value: 0.008). Regarding PCNA expression, it is notable to acknowledge the research conducted by Li and Zhouin et al. and by Song et al. [12,27]. The findings of the aforementioned studies indicate that a significant association exists between URG4 expression and the PCNA-labelled index in both epithelial ovarian cancer and gastric cancer [12,27]. Patients diagnosed with osteosarcoma displayed elevated levels of PCNA expression, which was positively correlated with heightened URG4 expression. Additionally, there is evidence supporting the potential of URG4 as a valuable prognostic indicator in individuals diagnosed with osteosarcoma, as those with heightened expression levels of this protein displayed decreased rates of survival [24]. Similar results were obtained in patients stratified by grade of histological differentiation, staging, lymph node involvement and depth of invasion (T1/T2 versus T3/T4). Lower URG4 expression was associated with longer survival in G1, G2 and G3 patients. In patients with stage I, II or III of the disease, higher survival was associated with lower URG4 expression. Low URG4 expression was also associated with better 5-year survival in patients with N0, N1 and N2 disease. Similar results were seen in patients with T1/T2 versus T3/T4 depth of invasion.

The results of our study are similar to those obtained by other authors. For example, in gastric cancer patients, a high expression of URG4 expression was also detected. Song et al. showed that the overexpression of URG4 could promote cell proliferation, while the down-regulation of URG4 may be connected with the inhibition of cell proliferation and tumour-forming potential [12]. Moreover, the results of their study demonstrated that URG4 can promote or inhibit cell growth, in part through the action of cyclin D1 [12]. In primary nasopharyngeal cancer tissue, the expression of URG4 was positively correlated with clinical stage, larger tumour size (T classification), lymph node involvement (N classification) and nasopharyngeal and distant metastasis. Zhang et al. [26] revealed that URG4 could be an independent biomarker for the prognosis of cervical cancer and a therapeutic target for patients with early-stage cervical cancer. Interestingly, URG4 may be an adverse prognostic factor in bladder cancer. The overexpression of URG4 increases the expression of anti-apoptotic factors such as Bcl-2 and FLIP and inhibits the expression of pro-apoptotic factors such as caspase-3. It also promotes resistance to apoptosis in bladder cancer cells by activating the NF-κB pathway [13]. In non-small cell lung carcinoma, URG4 expression is correlated with the expression of MMP-9 expression, which is positively correlated in various cohorts of human NSCLC specimens, and NF-κB-activated MMP-9 expression contributes to the URG4-induced invasiveness of NSCLC cell lines [15]. Moreover, the high expression of URG4 might be associated with the process of cancer angiogenesis. Xing et al. demonstrated that the high expression of URG4 was connected with the high expression of VEGFC, NF-κB transcriptional activity, the levels of phosphorylated IκB kinase (IKK) and IκB-α, and expression of TNFα, IL-6, IL-8 and MYC. In addition, the suppression of NF-κB activity in HCC cells abrogated URG4-induced NF-κB activation and angiogenic capacity [27]. The high expression of URG4 is associated with poor prognosis in many types of cancer. However, Aslan et al. demonstrated that the increased expression of URG4 in breast ductal carcinomas is significantly associated with good prognostic parameters [19].

In summary, the Cox regression model results demonstrated that URG4 exhibits a statistically significant association with reduced 5-year survival among individuals afflicted by colon adenocarcinoma. In this study, it was evidenced that the histological grade of the tumour and the immunohistochemical expression of URG4 were distinct prognostic parameters in the context of this malignancy. This study represents a novel contribution to the field, as it represents the initial evidence for the immunohistochemical expression of URG4 in colon adenocarcinomas within the demographic of the Polish population. Furthermore, it offers corroborative data supporting the prognostic utility of URG4 expression in patient populations segregated based on distinct clinical oncologic parameters. In this case, several significant factors were considered, including tumour grade, depth of invasion, PCNA expression, and staging.

## 5. Conclusions and Limitations of the Study

Our investigation constitutes the initial documented instance of URG4 identification in colon adenocarcinoma tissue using electron microscopy techniques, specifically the incorporation of the immunogold labelling method. The present investigation is subject to several constraints and shortcomings. It is plausible that selection bias may have been encountered in the present study due to the limited sample size that was used and the sole inclusion of patients from a single hospital. It is recommended to augment the sample size in subsequent investigations.

## Figures and Tables

**Table 1:** Characteristics of the patients (*n* = 138).

		*n* (Number of Cases)	%
Gender	Females	68	49.28
	Males	70	50.72
Age [years]	≤60 years	50	36.23
	61–75 years	50	36.23
	>75 years	38	27.54
	M ± SD	65.12 ± 12.66	
	Me (Q1–Q3)	65 (56–77)	
	Min—Max	33–89	
The degree of the histological differentiation	G1	32	23.19
	G2	67	48.55
	G3	39	28.26
Depth of invasion	T1	22	15.94
	T2	19	13.77
	T3	73	52.90
	T4	24	17.39
Regional Lymph Node involvement	N0	65	47.10
	N1	38	27.54
	N2	35	25.36
Location of tumour	Right part of the colon	72	52.17
	Left part of the colon	66	47.83
URG4 expression in non-cancerous tissue	Low	124	89.86
	High	14	10.14
Angioinvasion	No	38	27.54
	Yes	100	72.46
Immunohistochemical expression of proliferating cell nuclear antigen (PCNA)	Low	28	20.29
	High	110	79.71
Staging	I	33	23.91
	II	32	23.19
	III	73	52.90

**Table 2:** Association between the expression of URG4 protein and proliferating cell nuclear antigen (PCNA) (as dependent variables).

		The Immunoexpression Level of URG4				*p*-Value
		Low		High		
Proliferating cell nuclear antigen expression	Low	23	(16.67%)	5	(3.62%)	*p* < 0.001
	High	15	(10.87%)	95	(68.84%)	*p* = 0.044

**Table 3:** URG4 protein expression and clinicopathological features in patients with colorectal adenocarcinoma (as independent variables).

		The Immunoexpression Level of URG4				*p*-Value
		Low		High		
Age [Years]	≤60 years	15	(30.00%)	35	(70.00%)	*p* = 0.777
	61–75 years	12	(24.00%)	38	(76.00%)	
	>75 years	11	(28.95%)	27	(71.05%)	
Gender	Females	23	(33.82%)	45	(66.18%)	*p* = 0.103
	Males	15	(21.43%)	55	(78.57%)	
Grade of histological differentiation (G)	G1	24	(75.00%)	8	(25.00%)	*p* < 0.001
	G2	12	(17.91%)	55	(82.09%)	
	G3	2	(5.13%)	37	(94.87%)	
Depth of invasion (T)	T1/T2	23	(56.10%)	18	(43.90%)	*p* < 0.001
	T3/T4	15	(15.46%)	82	(84.54%)	
Regional Lymph Node involvement	N0	22	(33.85%)	43	(66.15%)	*p* = 0.276
	N1	9	(23.68%)	29	(76.32%)	
	N2	7	(20.00%)	28	(80.00%)	
Localisation	Left part of the colon	19	(26.39%)	53	(73.61%)	*p* = 0.753
	Right part of the colon	19	(28.79%)	47	(71.21%)	
Angioinvasion	Yes	25	(65.79%)	13	(34.21%)	*p* < 0.001
	No	13	(13.00%)	87	(87.00%)	
Expression of proliferating cell nuclear antigen	Low	23	(82.14%)	5	(17.86%)	*p* < 0.001
	High	15	(13.64%)	95	(86.36%)	
Staging of colon cancer (I, II, III)	I	17	(51.52%)	16	(48.48%)	*p* = 0.002
	II	5	(15.63%)	27	(84.38%)	
	III	16	(21.92%)	57	(78.08%)	

**Table 4:** Univariate and multivariate analyses of different prognostic parameters in patients with colon adenocarcinoma by means of Cox regression analyses.

Prognostic Parameter	Univariate Analysis			Multivariate Analysis		
	HR	95% CI	*p*-Value	HR	95% CI	*p*-Value
Gender Reference kategory: Female	1.017	0.684–1.511	0.935	–	–	–
Age	1.004	0.989–1.019	0.633	–	–	–
Staging	1.365	1.066–1.749	0.014	0.756	0.433–1.318	0.324
Grade of histological differentiation	2.758	2.051–3.708	<0.001	1.958	1.295–2.962	0.001
Depth of invasion	1.836	1.450–2.324	<0.001	1.278	0.880–1.856	0.198
Regional Lymph Node involvement	1.270	1.003–1.609	0.047	1.011	0.673–1.520	0.957
Localisation Reference category: Left	1.100	0.740–1.636	0.637	–	–	–
Immunohistochemical expression of URG4 in cancer tissue	4.296	2.427–7.605	<0.001	2.217	1.159–4.241	0.016
Angioinvasion Reference category: No	3.957	2.233–7.013	<0.001	1.003	0.446–2.256	0.994
Expression of proliferating cell nuclear antigen (PCNA) Reference category: Low	5.392	2.602–11.174	<0.001	1.592	0.534–4.743	0.404

## Data Availability

Data is contained within the article.

## References

[1] Douaiher J., Ravipati A., Grams B., Chowdhury S., Alatise O., Are C. (2017). Colorectal cancer-global burden, trends, and geographical variations. J. Surg. Oncol..

[2] Bray F., Ferlay J., Soerjomataram I., Siegel R.L., Torre L.A., Jemal A. (2018). Global cancer statistics 2018: GLOBOCAN estimates of incidence and mortality worldwide for 36 cancers in 185 countries. CA Cancer. J. Clin..

[3] Wong M.C.S., Huang J., Lok V., Wang J., Fung F., Ding H., Zheng Z.J. (2021). Differences in Incidence and Mortality Trends of Colorectal Cancer Worldwide Based on Sex, Age, and Anatomic Location. Clin. Gastroenterol. Hepatol..

[4] Arnold M., Sierra M.S., Laversanne M., Soerjomataram I., Jemal A., Bray F. (2017). Global patterns and trends in colorectal cancer incidence and mortality. Gut.

[5] Sawicki T., Ruszkowska M., Danielewicz A., Niedźwiedzka E., Arłukowicz T., Przybyłowicz K.E. (2021). A Review of Colorectal Cancer in Terms of Epidemiology, Risk Factors, Development, Symptoms and Diagnosis. Cancers.

[6] Fleming M., Ravula S., Tatishchev S.F., Wang H.L. (2012). Colorectal carcinoma: Pathologic aspects. J. Gastrointest. Oncol..

[7] Ho V., Chung L., Wilkinson K., Lea V., Lim S.H., Abubakar A., Ng W., Lee M., Roberts T.L., Chua W. (2023). Prognostic Significance of MRE11 Overexpression in Colorectal Cancer Patients. Cancers.

[8] Dayde D., Tanaka I., Jain R., Tai M.C., Taguchi A. (2017). Predictive and Prognostic Molecular Biomarkers for Response to Neoadjuvant Chemoradiation in Rectal Cancer. Int. J. Mol. Sci..

[9] Carpinetti P., Donnard E., Bettoni F., Asprino P., Koyama F., Rozanski A., Sabbaga J., Habr-Gama A., Parmigiani R.B., Galante P.A. (2015). The use of personalized biomarkers and liquid biopsies to monitor treatment response and disease recurrence in locally advanced rectal cancer after neoadjuvant chemoradiation. Oncotarget.

[10] Tufan N.L., Lian Z., Liu J., Pan J., Arbuthnot P., Kew M., Clayton M.M., Zhu M., Feitelson M.A. (2002). Hepatitis Bx antigen stimulates expression of a novel cellular gene, URG4, that promotes hepatocellular growth and survival. Neoplasia.

[11] Li W., Zhou N. (2012). URG4 upregulation is associated with tumor growth and poor survival in epithelial ovarian cancer. Arch. Gynecol. Obstet..

[12] Song J., Xie H., Lian Z., Yang G., Du R., Du Y., Zou X., Jin H., Gao J., Liu J. (2006). Enhanced cell survival of gastric cancer cells by a novel gene URG4. Neoplasia.

[13] Wu M., Chen J., Wang Y., Hu J., Liu C., Feng C., Zeng X. (2015). URGCP/URG4 promotes apoptotic resistance in bladder cancer cells by activating NF-κB signaling. Oncotarget.

[14] Chen L.C., Zhang H.Y., Qin Z.Y., Wang Y., Mao Y., Yao Y., Zhou L.F. (2014). Serological identification of URGCP as a potential biomarker for glioma. CNS Neurosci. Ther..

[15] Cai J., Li R., Xu X., Zhang L., Wu S., Yang T., Fang L., Wu J., Zhu X., Li M. (2015). URGCP promotes non-small cell lung cancer invasiveness by activating the NF-κB-MMP-9 pathway. Oncotarget.

[16] Dodurga Y., Eroğlu C., Seçme M., Elmas L., Avcı Ç.B., Şatıroğlu-Tufan N.L. (2016). Anti-proliferative and anti-invasive effects of ferulic acid in TT medullary thyroid cancer cells interacting with URG4/URGCP. Tumour Biol..

[17] Dodurga Y., Avcı C.B., Susluer S.Y., Satıroğlu Tufan N.L., Gündüz C. (2012). The expression of URGCP gene in prostate cancer cell lines: Correlation with rapamycin. Mol. Biol. Rep..

[18] Dodurga Y., Oymak Y., Gündüz C., Satıroglu-Tufan N.L., Vergin C., Cetingül N., Biray Avci C., Topçuoğlu N. (2013). Leukemogenesis as a new approach to investigate the correlation between up regulated gene 4/upregulator of cell proliferation (URG4/URGCP) and signal transduction genes in leukemia. Mol. Biol. Rep..

[19] Aslan F., Avcıkurt A.S. (2019). URG4 expression in invasive breast carcinoma and its relation to clinicopathological characteristics. Breast Cancer.

[20] Stoimenov I., Helleday T. (2009). PCNA on the crossroad of cancer. Biochem. Soc. Trans..

[21] Guzińska-Ustymowicz K., Pryczynicz A., Kemona A., Czyzewska J. (2009). Correlation between proliferation markers: PCNA, Ki-67, MCM-2 and antiapoptotic protein Bcl-2 in colorectal cancer. Anticancer Res..

[22] Kontomanolis E.N., Koutras A., Syllaios A., Schizas D., Mastoraki A., Garmpis N., Diakosavvas M., Angelou K., Tsatsaris G., Pagkalos A. (2020). Role of Oncogenes and Tumor-suppressor Genes in Carcinogenesis: A Review. Anticancer Res..

[23] Zhang X.D., Wang Y., Ye L.H. (2014). Hepatitis B virus X protein accelerates the development of hepatoma. Cancer Biol. Med..

[24] Liu Y., Xi Y., Chen G., Wu X., He M. (2020). URG4 mediates cell proliferation and cell cycle in osteosarcoma via GSK3β/β-catenin/cyclin D1 signaling pathway. J. Orthop. Surg. Res..

[25] Yu G., Meng Q., Zhang T., Zeng C., He B., Zhang S. (2016). URG4 expression is a novel prognostic factor for the progression of nasopharyngeal carcinoma and overall survival of patient. Onco. Targets. Ther..

[26] Zhang L., Huang H., Zhang L., Hou T., Wu S., Huang Q., Song L., Liu J. (2014). URG4 overexpression is correlated with cervical cancer progression and poor prognosis in patients with early-stage cervical cancer. BMC Cancer.

[27] Xing S., Zhang B., Hua R., Tai W.C., Zeng Z., Xie B., Huang C., Xue J., Xiong S., Yang J. (2015). URG4/URGCP enhances the angiogenic capacity of human hepatocellular carcinoma cells in vitro via activation of the NF-κB signaling pathway. BMC Cancer.

